# Analysis of Models of Doxorubicin-Induced Cardiomyopathy in Rats and Mice. A Modern View From the Perspective of the Pathophysiologist and the Clinician

**DOI:** 10.3389/fphar.2021.670479

**Published:** 2021-06-03

**Authors:** Ekaterina Yu Podyacheva, Ekaterina A. Kushnareva, Andrei A. Karpov, Yana G. Toropova

**Affiliations:** Almazov National Medical Research Centre, Ministry of Health of the Russian Federation, Saint-Petersburg, Russia

**Keywords:** anthracycline cardiomyopathy, doxorubicin-induced cardiotoxicity, oxidative stress, anthracycline drugs, doxorubicin

## Abstract

Today the pharmacological possibilities of treating cancer are expanding and as a result, life expectancy is increasing against the background of chemotherapy and supportive treatment. In the conditions of successful antitumor treatment, complications associated with its toxic effect on healthy tissues and organs began to come to the fore. Anthracycline cardiomyopathy was the first serious cardiovascular complication to draw the attention of oncologists and cardiologists around the world. Anthracycline drugs such as doxorubicin, epirubicin, idarubicin are still widely used in oncological practice to treat a wide range of solid and hematological malignancies. Doxorubicin-induced cardiomyopathy is closely associated with an increase in oxidative stress, as evidenced by reactive oxygen species (ROS) nduced damage such as lipid peroxidation, and decreased levels of antioxidants. Myofibrillar destruction and dysregulation of intracellular calcium are also important mechanisms, usually associated with doxorubicin-induced cardiotoxicity. Despite the abundance of data on various mechanisms involved in the implementation of doxorubicin-induced cardiotoxicity, a final understanding of the mechanism of the development of doxorubicin cardiomyopathy has not yet been formed. It poses the most significant challenges to the development of new methods of prevention and treatment, as well as to the unambiguous choice of a specific treatment regimen using the existing pharmacological tools. In order to resolve these issues new models that could reflect the development of the chemotherapy drugs effects are needed. In this review we have summarized and analyzed information on the main existing models of doxorubicin cardiomyopathy using small laboratory animals. In addition, this paper discusses further areas of research devoted to the development and validation of new improved models of doxorubicin cardiomyopathy suitable both for studying the mechanisms of its implementation and for the preclinical drugs effectiveness assessment.

## Introduction

According to the global statistics, the neoplasm is one of the main reasons for morbidity and mortality worldwide. Frequently encountered issues are seeking medical care in late stages of the disease and inaccessibility of diagnosis. Despite the active development of various approaches to cancer treatment, systemic chemotherapy remains the mainstay cancer treatment. The pharmacological development in the second half of the 20th century led to a stable annual increase in the life expectancy of cancer patients (Markham et al., 2020). However, within the conditions of successful cancer treatment, complications associated with its toxic effect on healthy tissues and organs began to come to the fore. Anthracycline cardiomyopathy was the first serious cardiovascular complication to have been noticed by oncologists and cardiologists worldwide.

Anthracyclines [doxorubicin (DOX), epirubicin, idarubicin] are still widely used as a chemotherapeutic agent in the treatment of solid and hematological malignancies. During the study of anthracyclines toxic effects, a dose-dependent nature of cardiovascular complications was discovered. Thus, the incidence of heart failure is approximately 3–5% with a cumulative dose of 400 mg/m^2^ of DOX, and the incidence of complications reaches 48% with an increase in the total dose of the resulting drug to 700 mg/m^2^ (Zamorano et al., 2016).

From a prognostic point of view, it is important to separate cardiovascular complications during treatment with anthracycline drugs according to the timing of the development. To date, acute cardiotoxicity has been distinguished, which developed at the time of chemotherapeutic treatment and manifested in the first year after completion of the anthracyclines administration. At the same time, chronic cardiotoxicity develops after the anticancer treatment (Hole et al., 2013). Сhronic cardiovascular complications are of great importance in the prognosis of cancer patients as they can lead to a significant deterioration in the quality and expectancy of life of those who have been successfully treated for malignant neoplasms.

Today the mechanisms of the damaging effect of anthracyclines on the myocardium are being actively studied in various experimental models. There is evidence supporting the role of oxidative stress, systemic inflammation, calcium metabolism disorders in the development of anthracycline cardiomyopathy (Zhang et al., 2012; Varela-López et al., 2019; Wallace et al., 2020). Despite a number of articles on this issue, there is no review devoted to the analysis of these models. Nonetheless, it is of vital importance when developing a model, which would comply with the modern approaches to the anticancer treatment.

## Modern Concepts of the Pathogenesis of Doxorubicin Cardiomyopathy

It is assumed that the main mechanisms of action of anthracyclines are as follows: they are easily captured by cells and localized in the nucleus. DOX has an ability to inhibit DNA biosynthesis by DNA intercalation. This drug has a high affinity for sites containing GC base pairs and forming hydrogen bonds between DOX and guanine; it leads to the appearance of ternary DOX-DNA-topoisomerase II complexes, which activate DNA damage responses and, ultimately, cause cell death (Santos and Goldenberg, 2018). Anthracyclines prevent the re-ligation of double-stranded DNA breaks, thus “poisoning” the enzyme. A disruption of this process, ATP-dependent cleavage of DNA strands, followed by a transfer of strands through the break and its ligation, leads to the DNA damage, which, in its turn, leads to mitotic and apoptotic cell death. Topoisomerase II (Top2) falls into two subtypes: Top2α and Top2β. The key mechanism of antitumor treatment with DOX is that it targets Top2α highly expressed in rapidly proliferating malignant cells. This leads to the suppression of the formation of the Top2-DNA cleavage complex, followed by the transcription arrest, which then results in the DNA damage and, ultimately, cell death (Minotti et al., 2004; Octavia et al., 2012; Santos and Goldenberg, 2018). Top2β is more common in resting cells such as cardiomyocytes and is associated with the development of cardiomyopathy. Top2β dysfunction results in the activation of the altered P53 tumor suppressor pathway, impaired calcium leads to the suppression processing, mitochondrial dysfunction, and increased apoptosis (McGowan et al., 2017).

**FIGURE 1:**
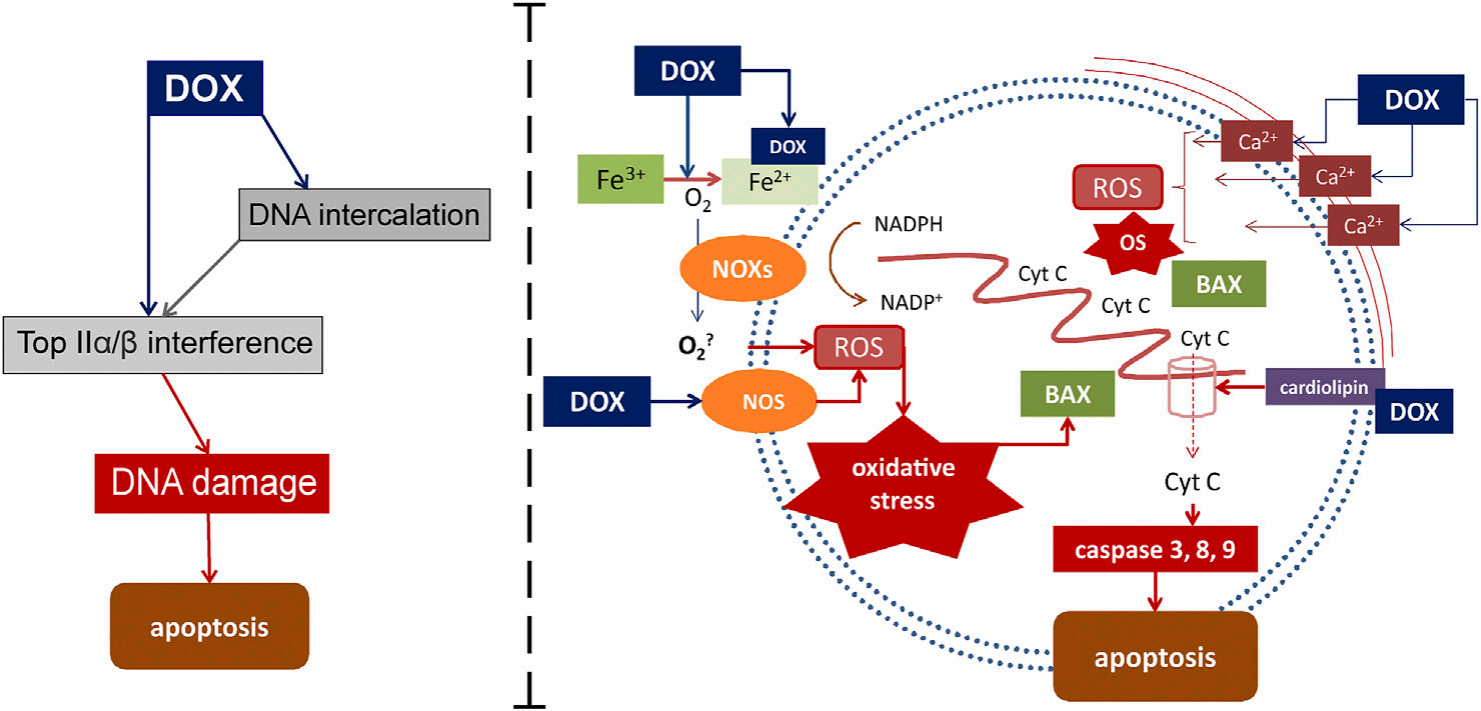
Mechanisms of DOX-induced cardiomyocyte damage. DOX targets Top2α/β. This leads to the suppression of the formation of the Top2-DNA cleavage complex, followed by the transcription arrest, which then results in the DNA damage and cell death. DOX also mediates apoptosis through interaction with Fe with subsequent active oxygen releasing and oxidative stress activation. Another way for oxidative stress activation is DOX-induced increasing of calcium ions releasing from the sarcoplasmic reticulum. Binding to cardiolipin DOX leads to mitochondrial dysfunction. Active cytochrome C releasing from mitochondrias to cytoplasm through the pores activates inner ways of apoptosis. BAX, Bcl-2-associated X protein; Cyt C-cytochrome C; DOX-doxorubicin; NOS-nitric oxide synthases; eNOS-endothelial NOS; NOXs-nicotinamide adenine dinucleotide phosphate oxidases; NADPH-nicotinamide adenine dinucleotide phosphate; ROS-reactive oxygen species.

Mechanisms that activate external and internal pathways of apoptosis are of key importance in the modern understanding of the pathogenesis of DOX cardiomyopathy. The quinone fragment of anthracyclines can undergo redox reactions with formation of excess reactive oxygen species (ROS) in the presence of oxidoreductive enzymes such as cytochrome P450 reductase, NADH dehydrogenase, and xanthine oxidase (May et al., 1980). Moreover, the ROS formation can be induced by the release of calcium ions from the sarcoplasmic reticulum of cardiomyocytes, which increases under the influence of DOX. In its turn, oxidative stress provokes the activation of the apoptosis regulators, such as BCl-2 associated X protein (BAX), which activates the release of cytochrome C through the pores in the mitochondrial membrane. This process induces the caspase cascade of activation of the external and internal pathways of apoptosis, which ultimately leads to the death of cardiomyocytes and the impairment of the contractile function of the heart. DOX-induced apoptosis targets not only cardiomyocytes, but also endothelial cells, as indicated by the activation of caspase and the degradation of the internucleosomal DNA (Lüscher and Barton, 2000; Octavia et al., 2012). In addition, cardiotoxicity associated with DOX administration is partially mediated by changes in the high-energy phosphate pool, endothelin-1 levels, and impaired adrenergic myocardial signaling (Renu et al., 2018). The current understanding of the role of oxidative stress in the development of DOX cardiomyopathy is presented in Figure 1.

Mitochondria are subcellular organelles which are most affected by DOX and show severe damage. This is conditioned by the fact that DOX, being a cationic drug, is retained in the inner mitochondrial membrane. Thus, an almost irreversible complex with cardiolipin is formed (Goormaghtigh et al., 1980). For the proteins in the electron transport chain to function properly, they must be bound to cardiolipin. However, it has been shown that as DOX disrupts the cardiolipin-protein interface, more superoxide (O2-) is produced (Kashfi et al., 1990; Schlame et al., 2000). These events obstruct mitochondrial (and therefore cellular) metabolism, since mitochondria generate more than 90% of the ATP used by cardiomyocytes (Ventura-Clapier et al., 2004). This functional disorder leads to ultrastructural pathological changes. In a rat model of chronic cardiomyopathy induced by DOX a general transition from erobic to anaerobic metabolism of the heart muscle was shown. This is directly related to a decrease in the oxidation of long-chain-fatty acids in the cardiac mitochondria and an increase in glucose metabolism. This shift in metabolism is a common feature of heart failure.

Starting from 1980, the first studies have showed that DOX has a strong affinity for iron (May et al., 1980). The doxorubicin-iron complex can induce lipid peroxidation by interacting with negatively charged membranes. Reduction of DOX in the presence of free iron starts the cycle of free radical formation (redox cycle). The interaction of the doxorubicinol metabolite with thiol groups of proteins exacerbates cell damage (Kappus et al., 1980; Xu et al., 2005). Under physiological conditions, free iron is insufficient to combine with DOX to such extent that it causes cardiomyopathy because the free iron content in most cells, including cardiomyocytes, is very low. More recent studies have shown that the effect of DOX on iron metabolism is mediated through proteins that sequester and bind intracellular iron. One of those mechanisms involves a formation of complexes of the doxorubicinol metabolite with the Fe-S group of cytoplasmic aconitase/IRE-BP (the iron-responsive element-binding proteins). This increases the stability of transferrin mRNA and prevents the translation of iron sequestration proteins (Minotti et al., 1998; Minotti et al., 2004). The subsequent decrease in IRE-BP leads to an increase in free iron, which can maintain the cycle of free radical generation. Thus, impaired iron sequestration remains a critical component of the DOX-induced cardiotoxicity (Nitobe et al., 2003).

NADPH is another enzyme that promotes formation of free radicals in the redox cycle (Santos and Goldenberg, 2018). It is a group of enzymes associated with the plasma membrane that serves as a source of ROS. Nicotinamide adenine dinucleotide phosphate (NADPH) oxidases (NOX) help convert the quinone moiety to a semi-quinone radical that can react with oxygen to form hydrogen peroxide. NOX can be activated by many stimuli, including TNF-α, and plays a key role in cardio remodeling (Santos and Goldenberg, 2018). In addition, it has been found that Nox 2 is a major mediator of NOX-derived ROS.

Nitric oxide (NO) is also a major contributor to oxidative stress caused by DOX. NO is a vasodilator that mediates heartbeats under normal homeostatic conditions. An increased level of NO is observed in DOX-induced cardiotoxicity due to NOS (NO-synthase) isoforms, namely endothelial NOS, inducible NOS, and neuronal NOS (Nebigil and Désaubry, 2018). DOX captures electrons from NADPH by direct binding of the eNOS reductase domain, and thatcontributes to the superoxide formation.

It is generally accepted that DOX-induced oxidative stress activates apoptotic signaling leading to apoptosis of cardiomyocytes (Nitobe et al., 2003). This involves both external and internal pathways of apoptosis (Papadopoulou et al., 1999). In the DOX model, oxidative stress activates HSF-1, which produces more Hsp25. Hsp25 stabilizes p53 and increases the production of pro-apoptotic proteins (Vedam et al., 2010; Octavia et al., 2012). These mechanisms play a significant role in the development of both acute and chronic cardiotoxicity. Liu et al. demonstrated that overexpression of Hsp27 also prevents DOX-induced apoptosis and myocardial dysfunction due to the protective role of Hsp27 in regulating oxidative stress responses and maintaining mitochondrial function (Liu et al., 2007). Hsp27 is known for its cardioprotective action against ischemia/reperfusion injury. Overexpression of Hsp10 and Hsp60 similarly leads to an increase in post-translational modification of Bcl-2 proteins, shifting the balance towards the transmission of antiapoptotic signals.

DOX-induced cardiotoxicity is also associated with increased intracellular calcium levels. Dysregulation of intracellular calcium concentrations results in the ROS formation. DOX causes a release of calcium from the sarcoplasmic reticulum by increasing the likelihood of the channel transition to an open state (Holmberg and Williams, 1990). Saeki et al. suggested that the ryanodine channel has multiple binding sites for DOX, and that binding occurs regardless of whether the channel is open or closed (Keung et al., 1991; Saeki et al., 2002). Most of the intracellular calcium in cardiomyocytes is present in the sarcoplasmic reticulum. Oxidative stress can cause calcium leakage, activation of calpain, and cleavage of caspase-12.

Caspase-12 activates apoptotic pathways, and its activation depends on the dysregulation of calpain, which sends distress signals from the sarcoplasmic reticulum (Octavia et al., 2012). Calpains are proteases activated by calcium. Calpains have been found to degrade titin, which is a large protein and a key component of the cardiac sarcomere that helps maintain cardiac contractility. Therefore, prevention of calpain activity helps maintain contractility after exposure to DOX (Santos and Goldenberg, 2018). It has also been found that DOX increases the sensitivity of mitochondria to intracellular calcium. A study in rats has shown that the mitochondria of heart cells treated with DOX have a reduced ability to retain calcium (Octavia et al., 2012).

Endothelin-1 (ET-1) signaling increases cell survival signaling in cardiomyocytes, which explains its activation in heart failure (Suzuki and Miyauchi, 2001). The expression of ET-1 and the expression of its receptors are increased in the myocardium of rats with congestive heart failure, in patients with idiopathic dilated cardiomyopathy and in patients treated with DOX (Picard et al., 1998; Pieske et al., 1999; Sayed-Ahmed et al., 2001). In the vasculature the activity of ET-1 is balanced by the action of NO. However, in patients treated with DOX, NO production is impaired (May et al., 1980; Miller et al., 1993; Li et al., 2006; Nakahara et al., 2018).

Despite the abundance of data on various mechanisms involved in the implementation of DOX-induced cardiotoxicity, a final understanding of the mechanism of the DOX cardiomyopathy development has not yet been formed. This is what causes difficulties in working out new methods of prevention and treatment, as well as in the unambiguous choice of a specific treatment regimen using the existing pharmacological tools.

## Anthracycline Cardiomyopathy Models

The review is based on the results of studies from articles and reviews published for the last 40 years. We have conducted a literature search using publicly available databases (PubMed, Google Scholar, Europe PubMed Central). All publications used while preparing this review are written in English, published in peer-reviewed journals, and contain original data on the experimental models including basic animal characteristics.

Both large and small laboratory animals are used to simulate the damaging effect of anthracycline antibiotics. Rabbits were the first animals to experience the chronic cardiotoxic effects of DOX. Maral et al. and later Jaenke have described histological and ultrastructural changes in the myocardium of rabbits treated with DOX for 3–4 months (Maral et al., 1967; Jaenke, 1974). These researchers have reported that the incidence and severity of cardiomyopathic lesions were the highest in the left ventricular wall and interventricular septum, intermediate in the atria, and the lowest in the right ventricular wall. Nephropathy (glomerular vacuolation, tubular injury, deposition of protein casts in the tubular lumen, and clinical and laboratory evidence of anephrotic syndrome with hypercholesterolemia) is also a common and important side effect of chronic DOX-induced toxicity in rabbits and other rodents. Such lesions do not develop in people treated with these DOX (Herman et al., 1985; Herman and Ferrans, 1997).

The main advantages of large laboratory animals, such as pigs, monkeys, dogs, include the similarity of their cardiovascular system anatomical structure with that of humans, similar pathological changes in the myocardium that occur during the administration of anthracyclines, as well as the possibility of conducting a wide range of functional studies characterizing the work of the heart (Herman and Ferrans, 1997). However, the large animal model has significant drawbacks, including the amount of medication needed and the technical difficulties that arise while blood sampling and performing multiple intravenous injections of DOX (anterior vena cava) (Herman and Ferrans, 1983; Herman and Ferrans, 1997). In addition, achieving a cardiotoxic effect requires a larger amount of anticancer drug and a longer period of time from the beginning of exposure to the development of signs of cardiotoxicity (Robert, 2007). These disadvantages make experiments on large models less productive and significantly more expensive.

The need for small laboratory animal’s models arose when it became necessary to study toxic properties of a number of anthracycline analogs. A model that would allow to study drugs on a fairly large sample of animals for a relatively short period of time was required, which, at the same time, would let assess the degree of myocardial damage and the effectiveness of potentially cardioprotective therapy. The most commonly used models that meet the above criteria are laboratory mice and rats.

### Rat Models of Anthracycline Cardiomyopathy

Mettler et al. first reported in 1977 that CDF rats (Fischer) treated with DOX 1–2 mg/kg per week for 10–14 weeks showed signs of congestive heart failure. Histological changes in these animals included vacuolization and degeneration of myocytes, interstitial edema and mild fibrosis (Mettler et al., 1977). In search of the best rat model Herman et al. in 1985 compared the severity of chronic DOX cardiotoxicity in adult male rats with spontaneous hypertension and in genetically related normotensive Wistar-Kyoto rats (Herman et al., 1985). Heart lesions were noted in both strains of rats. However, at a dose level of 0.25–1 mg/kg per week for 12 weeks, spontaneously hypertensive rats were significantly more sensitive to the cardiotoxic effects of DOX than normotensive rats. The mechanism of this hypersensitivity has not been determined. Because of their sensitivity and high degree of reproducibility of heart lesions, rats with spontaneous hypertension are more suitable than other rat strains as small animal models for studying anthracycline cadiotoxicity (Herman and Ferrans, 1997). However, this model reflects only a narrow proportion of patients with severe arterial hypertension who have undergone chemotherapy.

In general, models can be divided into short-term and long-term ones, depending on the injection protocols (Robert, 2007; Nakahara et al., 2018). In short-term rat models animals receive a single dose of DOX (10–30 mg/kg) intravenously (Todorova et al., 2012). Other short-term models use 2–3.4 mg/kg of DOX every other day, six times intraperitoneally (the total dose is 12–20 mg/kg) (Zbinden et al., 1978; Lanza et al., 1989; Carvalho et al., 2010; Razmaraii et al., 2016; Cappetta et al., 2018; Aygun and Gul, 2019; Barış et al., 2019). Long-term rat models typically use 1–5 mg/kg every week for 2–12 weeks with a cumulative dose of 3–25 mg/kg (Villani et al., 1991; Sacco et al., 2001; Sayed-Ahmed et al., 2001; Rahimi Balaei et al., 2010; Hydock et al., 2012; Hole et al., 2013; Toblli et al., 2014; Kang et al., 2017; Medeiros-Lima et al., 2019; Wang et al., 2019; Chakouri et al., 2020; Sharma et al., 2020). Thus, a short-term high-dose injection model would be suitable for assessing acute cardiotoxicity, while a long-term low-dose model would be suitable for assessing chronic cardiotoxicity.

Recent studies often research models that use DOX 6 times every other day at 2.5 mg/kg (Merlet et al., 2013; Lončar-Turukalo et al., 2015; Cappetta et al., 2017a; Ahmad et al., 2017; Cappetta et al., 2017b; Elhadidy et al., 2020). Histological examination showed that this injection protocol results in irregularly spaced myocardial fibers with inflammation, edema, and leukocyte cell infiltration. Other models use 1 mg/kg of DOX for 10 days and 1.25 mg/kg of DOX 4 times a week for 4 weeks (Medeiros-Lima et al., 2019; Aykan et al., 2020). The results show that DOX significantly reduced body, heart and left ventricular weight one week after stopping DOX. This trend persists after 2 and 4 weeks. Interstitial fibrosis of the left ventricle progressively increases after the end of DOX administration. Cardiac troponin I levels increase significantly one and two weeks after the end of DOX administration increase even more after four weeks.

In a study aimed at achieving DOX-induced chronic cardiomyopathy, rats received 2.15 mg/kg of Dox for 3 weeks 7 times (Ivanová et al., 2012). The researchers noted that animals treated with DOX had lower body weight compared to the control animals (treated with saline). However, the total heart weight as well as the left ventricular weight did not change significantly. Eight weeks after the end of the treatment systolic blood pressure increased in DOX-treated rats compared to the control animals, whereas there was no difference in heart rate. Electron microscopic examination of the heart shows that DOX induces heterogeneous subcellular changes characterized by degeneration and/or loss of myofibrils, as well as cytoplasmic vacuolization of cardiomyocytes. These changes are known as structural markers of DOX cardiotoxicity. Structural disorganization of the extracellular space of the heart is also observed, which is manifested in an increase in the density of extracellular matrix proteins. Simultaneously, ultrastructural analysis shows the presence of damaged capillary endothelial cells. The damage is characterized by accidental disruption of plasmalemmal integrity, damage to mitochondria, changes in cytoplasmic density, number of ribosomes, pinocytic vesicles, and random disorganization and accumulation of chromatin, which indicates activation and dysfunction of endothelial cells. Moreover, in the hearts of rats treated with DOX the following has been noted: an increased accumulation of interstitial collagen fibers; presence of macrophages, vacuoles forming large membrane-bound spaces, Cajal cell processes and proliferating fibroblasts. All these changes appear in the fourth week after the end of the DOX administration and worsen by the eighth week (Ivanová et al., 2012).

Thus, there is still no single universal model that creates DOX chronic cardiotoxicity in rats. Table 1 presents data on the use of rat models of DOX cardiomyopathy (Table 1).

**TABLE 1:** Data on the modeling of doxorubicin cardiomyopathy in rats.

No. References	Sex, age/body weight	Dose of DOX	Administration frequency	Route of administration	Toxicity assessment
Zbinden et al. (1978)	Female 150 g	0.1 ml/10 g body weight	5 days per week	IP	ECG, histology
Lanza et al. (1989)	Female Sprague Dawley 120–130 g	3 mg/kg → 9 mg/kg	every 3rd day	IP	ECG, creatinine, glucose, sodium, potassium, calcium, lactate dehydrogenase, alkaline phosphatase, transaminase
Villani et al. (1991)	Female CD 120–130 g	3 mg/kg → 12 mg/kg	Once a week for 4 weeks	IP	ECG, histology
Sacco et al. (2001)	Male Sprague–Dawley 27–29 days old	1.5 mg/kg → 7.5 mg/kg	Once a week for five consecutive weeks	IP	ECG
Rahimi Balaei et al. (2010)	Male Wistar 200–230 g	1.25 mg/kg → 20 mg/kg	Four times per week during first month	IP	ECG, troponin, histology
Hydock et al. (2012)	Female Sprague-Dawley	1 mg/kg → 15 mg/kg; 2.5 mg/kg → 15 mg/kg	15 consecutive days; six consecutive weeks	IP	ECHO
Ivanová et al. (2012)	Male Wistar 10 weeks	2.15 mg/kg → 15 mg/kg	7 times per week for 3 weeks	IP	ECHO, histology
Todorova et al. (2012)	Male Sprague-Dawley	12 mg/kg	Single dose	IP	ALT, ALB, ALP, AMY, CA++, CRE, GLOB, GLU, PHOS, K+, NA+, TBIL, TP, BUN
Hole et al. (2013)	Male Wistar 200 ± 2 g	2 mg/kg → 10 mg/kg	5 consecutive days	IP	ECHO, H_2_O_2_, troponin
Merlet et al. (2013)	Male, Sprague-Dawley 225–250 g	2.5 mg/kg → 15 mg/kg	Six equal injections over a period of two weeks	IP	ECHO
Toblli et al. (2014)	Male SHR-SP Three-week old	3, 4, or 5 mg/kg	once a week for 6 weeks	IP	ECHO, histology, TBARS, MDA, glutathione, catalase, Cu,ZnSOD, glutathione peroxidase
Lončar-Turukalo et al. (2015)	Male Wistar 300 ± 10 g	2.5 mg/kg → 15 mg/kg	Six equal injections over a period of two weeks	IP	ECHO, histology, troponin
Razmaraii et al. (2016)	Male Wistar180–220 g	2 mg/kg → 12 mg/kg	6 equal doses per 48 hours over a period of 12 days	IP	ECHO, histology
Nakahara et al. (2018)	Male, Sprague-Dawley 8 weeks	Rat short-term model: 2–3.4 mg/kg → 12–20 mg/kg.rat long-term model: 1–5 mg/kg → 3–25 mg/kg	Six equal injections over a period of two weeks	IP	ECHO, histology, troponin
Ahmad et al. (2017)	Male Wistar 150–180 g	2.5 mg/kg → 15 mg/kg	Six equal injections over a period of two weeks	IP	ECG, troponin, TNF-a, lactate dehydrogenase, creatine kinase, cardiac thiobarbituric acid reactive substance, glutathione
Cappetta et al. (2017b)	Male Fischer 3 months	2.5 mg/kg → 15 mg/kg	Six equal injections over a period of two weeks	IP	ECHO, histology
Kang et al. (2017)	Male Wistar 250.4 ± 4.3 g	2.5 mg/kg → 10 mg/kg; 3 mg/kg→ 12 mg/kg; 3.5 mg/kg → 14 mg/kg; 4 mg/kg → 16 mg/kg	Weekly intervals for up to 4 weeks	IP	ECHO, histology, troponin
Medeiros-Lima et al. (2019)	Male Sprague-Dawley 12 weeks	1 mg/kg →10 mg/kg	10 times every day	IP	ECHO, histology, troponin
Elhadidy et al. (2020)	Male 220–250 g	2.5 mg/kg → 15 mg/kg	Six equal injections over a period of two weeks	IP	ECG, histology
Wang et al. (2019)	Male Sprague-Dawley 220–250 g	4 mg/kg → 12 mg/kg	3 times: 1, 6, 11 days	IP	ECHO
Aygun and Gul (2019)	Male Wistar 225–280 g	18 mg/kg	For three days in the study	IP	ECG
Barış et al. (2019)	Male Sprague–Dawley 300–400 g	25 mg/kg	For 3 days; on 12th, 13th, and 14th days	IP	ECHO, histology
Aykan et al. (2020)	Male, Wistar 200–300 g	1.25 mg/kg → 20 mg/kg	4 days a week during for 4 weeks	IP	ECHO, histology
Sayed-Ahmed et al. (2001)	Male Sprague-Dawley 7–9 weeks	2 mg/kg → 8 mg/kg	4 times: once a week for 4 weeks	IP	Histology, troponin
Chakouri et al. (2020)	Male Wistar 4 weeks	Three cumulative doses: 7.5, 10 or 12.5 mg/kg	Once a week for 6 weeks	Intravenous injection (tailvein)	ECHO, histology

ALT, alanine aminotransferase; ALB, albumin; ALP, alkaline phosphatase; AMY, amylase; BUN, urea nitrogen; Cu, ZnSOD, Cu, Zn superoxide dismutase; CA++, total calcium; CRE, creatinine; ECG, electrocardiography; ECHO, echocardiography; GLOB, globulin; GLU-glucose; IP, intraperitoneally injection; K+, potassium; MDA, malondialdehyde; NA+, sodium; PHOS, phosphorus; TBARS, thiobarbituric acid reactive substances; TBIL, total bilirubin; TP, total protein.

### Mouse Models of Anthracycline Cardiomyopathy

The first mouse DOX cardiomyopathy model was presented by Bertazzoli et al. (1979). Two administration regimens were studied: 1.4 and 4 mg/kg twice a week up to 10 administrations in total. After a low dose administration cardiomyocyte vacuolization was detected. However, with a higher dose of DOX more severe pathological changes were noted, such as atrophy, myofibrils loss, necrosis with connective tissue proliferation, etc. In addition, significantly higher involvement of other tissues and organs (kidneys, spleen and liver) was discovered. Initial attempts to establish DOX cardiomyopathy models predominantly used intravenous (IV) administration through the tail vein (Forssen and Tokes, 1981; Kanter et al., 1985; van der Vijgh et al., 1988; van Acker et al., 1996). However, this method was not convenient in the context of handling of a large number of animals and it also required specialized technical skills.

Later, intraperitoneal (IP) method of DOX administration was tested and accepted into practice (Mostafa et al., 2000; Abd-Allah et al., 2002; Li et al., 2006; Neilan et al., 2006; Pei et al., 2016). Johansen compared DOX pharmacokinetic properties using two different types of administration: IV and IP. (Johansen, 1981). These experiments showed that plasma drug concentration after 24 hours was similar in the compared groups. He also evaluated DOX concentration in different tissues—that of muscles, kidneys, liver and heart. It was shown that after 24 hours the drug concentration in tissues was also similar. This method of administration has been widely put into practice and is now the most commonly used method for DOX cardiomyopathy modeling. However, inaccurate drug administration still poses risk of damaging organs.

The year 2019 saw the emerging of a novel method of DOX administration. It involved using slow-released subcutaneous pellets with a 5 mg/kg total cumulative dose, which is released over a period of 21 days (∼0.25 mg/kg/day) (Allen et al., 2019; Naresh et al., 2020). Myocardial changes after the cumulative dose achievement were similar to those after IP administration. Therefore, this method is very innovative for developing a chronic DOX cardiomyopathy model. Nowadays other measures are taken to verify DOX cardiomyopathy besides histological changes, such as evaluation of other cardio-markers, includingtroponin, NT-proBNP and assessment of systolic function by echocardiography (Xujie et al., 2012; Sahu et al., 2016; Pillai et al., 2017; Vandenwijngaert et al., 2017; Ye et al., 2020). Information about features of mouse models is summarized in Table 2.

**TABLE 2:** Data on the modeling of doxorubicin cardiomyopathy in mouse.

No. References	Sex, age	Dose of DOX	Administration frequency	Route of administration	Toxicity assessment
Forssen and Tokes (1981)	—	5 mg/kg → 20 mg/kg	Weekly	IV	Histology
	—				
Forssen and Tokes (1981)	—	5 mg/kg → 40 mg/kg	Weekly	IV	Histology
	—				
Kanter et al. (1985)	Male	4 mg/kg (cumulative dose is unknown)	Once a 10 days	IV	Histology
	∼35 days				
van der Vijgh et al. (1988)	Female	2 mg/kg (cumulative dose is unknown)	12 times with treatment-free interval after the 4th injection	IV	Histology
	—				
van Acker et al. (1996)	Male	4 mg/kg → 24 mg/kg	Weekly	IV	Implantable ECG analog, histology
	—				
Mostafa et al. (2000)	Male/female	15 mg/kg	Once	IP	CPK, histology
	—				
Abd-Allah et al. (2002)	Male	15 mg/kg	Once	IP	CK
	—				
Li et al. (2006)	Male	20 mg/kg	Once	IP	ECHO, histology
	9–10 weeks				
Neilan et al. (2006)	Male	20 mg/kg	Once	IP	ECHO, invasive haemodynamic assessment, histology
	8–10				
Xujie et al. (2012)	Male	2 mg/kg → 20 mg/kg	Every other day for 8 days, then once a week	IP	ECHO, histology
	6–8 weeks				
Xujie et al. (2012)	Male	2.5 mg/kg → 15 mg/kg	Every other day for 12 days	IP	ECHO, histology
	6–8 weeks				
Pei et al. (2016)	Male	15 mg/kg	Once	IP	ECHO
	14–18 weeks				
Sahu et al. (2016)	Male	5 mg/kg → 15 mg/kg	Every 5 days	IP	CK-MB, LDH, ALS, AST, histology
	7–8 weeks				
Pillai et al. (2017)	Male	5 mg/kg → 15 mg/kg	Every 15 days	IP	ECHO
	—				
Vandenwijngaert et al. (2017)	—	20 mg/kg	Once	IP	ECHO
	8 weeks				
Vandenwijngaert et al. (2017)	Male/female	2 mg/kg → 24 mg/kg	Weekly	IP	ECHO
	8–12 weeks				
Hullin et al. (2018)	Male	15 mg/kg	Once	IP	ECHO
	8–10 weeks				
Hullin et al. (2018)	Male	4 mg/kg → 20 mg/kg	Weekly	IP	ECHO
	8–10 weeks				
Bai and Wang (2019)	Male	5 mg/kg	—	IP	Troponin
	12–14 weeks				
Gioffré et al. (2019)	Female	4 mg/kg → 24 mg/kg	3 times a week	IP	ECHO, troponin
	10 weeks				
Allen et al. (2019)	Female	8 mg/kg → 24 mg/kg	Weekly	IP	MRI
	10–12 weeks				
Allen et al. (2019)	Male	Cumulative 15 mg/kg	—	Subcutaneous pellets	Histology
	8 weeks				
Allen et al. (2019)	Male	Cumulative 25 mg/kg	—	Subcutaneous pellets	Histology
	8 weeks				
Naresh et al. (2020)	—	Cumulative 25 mg/kg	—	Subcutaneous pellets	MRI, histology
	15–16 weeks				
Mizuta et al. (2020)	Male	20 mg/kg	Once	IP	ECHO, CK-MB, LDH, histology
	9–10 weeks				
Hu et al. (2020)	Male	15 mg/kg	Once	IP	ECHO, troponin, CK
	10 weeks				
Sabatino et al. (2020)	Male	4 mg/kg → 20 mg/kg	Weekly	IP	ECHO, troponin, NT-proBNP, histology
	8 weeks				
Peres Diaz et al. (2020)	Male	10 mg/kg	Once	IP	Histology
	8 weeks				
Ye et al. (2020)	Male	15 mg/kg	Once	IP	ECHO, LDH, CK-MB, histology
	6 weeks				

ALS, alanine aminotransferase; AST, aspartate aminotransferase; CPK, creatine phosphokinase; CK-MB, creatine kinase myocardial bound; ECG, electrocardiography; ECHO, echocardiography; IP, intraperitoneally injection; IV, intravenous injection; LDH, lactate dehydrogenase; MRI, cardiac magnetic resonance imaging.

In current experimental practice mouse models are used either for acute (with one-time high dose administration) or chronic (with serial administration until the cumulative dose has been achieved) DOX cardiomyopathy modeling (Hullin et al., 2018; Bai and Wang, 2019; Gioffré et al., 2019; Hu et al., 2020; Peres Diaz et al., 2020). It is worth noting that an important criterion for the model quality is the animal survival rate. It is an important factor for the dynamic evaluation and potential cardioprotective drugs examination. Mizuta et al. showed that a single dose administration with 30 mg/kg leads to a high mortality rate during the first 6 days, that is why the dose had to be decreased to 20 mg/kg to continue the dynamic research (Mizuta et al., 2020).

Sabatino et al. revealed that with 4 mg/kg DOX administration until the achievement of the cumulative dose of 20 mg/kg a significant fibrosis, fibrin exhaustion (92%), myocardial fibers disorganization (67%), tortuosity (100%) and myocardial vacuolization (100%) were observed during 5 weeks (Sabatino et al., 2020). As shown in Table 2 there is still no common experimental chronic DOX cardiomyopathy model with good reproducibility. All models vary in antitumor drug dose and administration frequency. Therefore, this fact gives us no opportunity to compare the results of different experiments run in different conditions.

## Discussion

In experimental practice various types of laboratory animals are widely used to create models of DOX cardiomyopathy. However, studies on small laboratory animals (rats and mice) are dominant. At the same time, the criteria for assessing myocardial damage while administering DOX do not differ significantly and include: functional assessment of the myocardium using echocardiography, assessment of markers of myocardial damage (troponin, natriuretic peptide), as well as histological examination with an assessment of the degree of damage and development of fibrosis.

It should be noted that models generated in rats and mice do not show a drastic contrast in the severity of morphological changes in the myocardium. The processes of inflammation, myocardial fibrosis, disorganization and vacuolization of myocardial fibers in both species are widely represented in the myocardium while administering DOX. However, in a number of studies on mice histological examination has not been carried out due to a small amount of myocardial tissue, which was used for other experimental purposes, such as analysis of the expression of proteins, micro-RNA, etc. This circumstance can be considered a significant limitation while selecting mice for modeling DOX cardiomyopathy.

There is a number of studies in which modeling of DOX cardiomyopathy is performed simultaneously with modeling the tumor process. In this case, the choice of mice for research seems to be more rational due to the fact that these animals are genetically more pure lines in comparison with rats. However, the rat is a preferred animal for generating a breast cancer model as simulated tumors in rats provide an adequate similarity to human neoplasms. In particular, models of breast cancer in rats in most cases are hormone sensitive, while in mouse models tumor growth does not depend on estrogen activity (Mollard et al., 2011).

It is also worth noting that in terms of calculating the administered dose of DOX per kilogram of body weight and taking into account the anthropometric characteristics of animals, experiments on mice seem to be more economical in terms of the volume of the drug administered. However, the small size of animals can present difficulties in the selection and dilution of the required single dose of DOX and in its administration. The latter issue has been successfully resolved in recent years by approbation of models with intraperitoneal injection, which demonstrated good bioavailability of the drug and sufficient development of changes in the myocardium to recognize the model of DOX cardiomyopathy as effective (Zbinden et al., 1978).

It should be noted that with the currently available large number of experimental models of DOX cardiomyopathy using small laboratory animals (mice and rats), their prevailing majority is characterized by similar approaches to verification of cardiomyopathy, including histological, biochemical, electron microscopic studies, changes in the functioning of the myocardium. In the above mentioned studies there were no differences in pathological changes in the modeling of DOX cardiomyopathy between female and male rats/mice. Meanwhile, the described diversity of studies is characterized by the use of animals of different genetic status (belonging to the stock, line), as well as the absence of a unified approach to the presentation of data concerning the characteristics of animals. For instance, in some studies, sex and weight of animals are specified as the main characteristics of animals, while in other studies their sex and age are specified. In addition, the presented studies differ in the doses of DOX used. Given the proven role of the genetic background in the formation of phenotypic differences that can influence the results of the experiment (Simon et al., 2013); the complexity of comparing the doses of DOX used with the weight characteristics of animals (hence, the calculation of the cumulative dose of the drug); as well as, in some cases, the lack of the evaluation criteria reflecting the development of pathogenetic links (for example, oxidative stress) and the physical state of animals (reflecting the severity of myocardial damage), we can assume one of the probable reasons for the “failure” of pathogenetically grounded experimental treatment in a clinical setting.

It is known that some of the drugs entering clinical development do not confirm their effectiveness previously demonstrated in laboratory conditions (Pound and Ritskes-Hoitinga, 2018; Ferreira et al., 2020). This situation also applies to drugs intended for a preventive or pathogenetic effect on the course of DOX cardiomyopathy (Weidemann and Strotmann, 2006; Vincent et al., 2013; Lipshultz et al., 2014; Abushouk et al., 2017; Kim et al., 2019; Wang et al., 2019; Sharma et al., 2020). Possible reasons for such low transferability can be considered the presence of species differences between humans and laboratory animals, as well as the difficulty in developing an adequate experimental model of the disease. Taking into account the expansion of pharmacological possibilities of cancer treatment, and, as a result, the increase in the life expectancy due to chemotherapy and supportive treatment, it becomes necessary to search for mechanisms that would implement the development of those effects of chemotherapeutic drugs that have not been previously encountered. In order to resolve these issues new models are needed that could reflect these effects.

Ideally, the experimental model should meet certain criteria: the similarity of morphological, biochemical and functional characteristics at various levels of integration of the organism (systemic, organ, cellular, subcellular, molecular); the similarity of the mechanism of development (pathogenesis) of the disease and its phenotypic manifestations (biochemical, functional and morphological signs); the generality of the principles of treatment for humans and the animal model. In order to select the optimal dose of DOX administration to an animal, we must have a good understanding of the clinical symptoms developing in people who received chemotherapy treatment. Subclinical cardiotoxicity in humans is usually defined by cardiac echocardiography as clinically asymptomatic left ventricular systolic dysfunction with a fall in ejection fraction (EF) of more than 10% to an EF value of <50%. If asymptomatic reductions in EF are prospectively detected within the first 12 months of the follow-up after chemotherapy, this indicates early cardiotoxicity (McGowan et al., 2017). The analysis for cardiac troponin also plays an important role in the determination of various heart diseases. In the context of chemotherapy, elevated troponin can quantify both cardiomyocyte apoptosis and myofibril degradation and predict cardiotoxicity, defined as a drop in EF, as well as serious adverse cardiovascular complications. In view of all above mentioned aspects, it can be assumed that when creating an actual model of DOX cardiomyopathy in animals, in this case in rodents, there should be a decrease in the EF by more than 10%, confirmed by echocardiographic studies; an increase in the level of cardiac troponin, which characterizes damage to cardiomyotes; and most importantly, the long-term survival of animals and their satisfactory physical condition.

The most commonly used dose of DOX in rats ranges from 1 to 2.5 mg/kg, which constitutes a cumulative dose of 10–20 mg/kg. The results of most studies demonstrate that DOX significantly reduces body and heart weight, increases cardiac troponin levels, and results in the accumulation of interstitial collagen fibers in the left ventricle, manifested at the end of administration and aggravated by the 4th or 8th week. Of all the studies described in the review concerning the modeling of DOX cardiomyopathy in rats, it can be assumed that the optimal cumulative doses of DOX administration providing clinical manifestations confirmed by echocardiographic and biochemical studies, comparable to people who have undergone chemotherapy and ensuring long-term survival of animals, start from 10 mg/kg and less. It can be assumed that the experimental model characterized by changes that reflect the main pathogenetic links of this disease, as well as the integrated approach to the verification of the model will allow to consider it on the one hand, as a basic model for studying the mechanisms underlying the development of DOX cardiomyopathy, and, on the other hand, as an adequate model for preclinical assessment of pathogenetically based treatment.

In this review, we have summarized and analyzed the main models of DOX cardiomyopathy using small laboratory animals (rats and mice). We have also proposed approaches to improve the model, taking into account the species differences between humans and laboratory animals, biochemical and histological differences, the severity of changes in the morphology and functional characteristics of the myocardium.
