# Supplementary material for: Analysis of Models of Doxorubicin-Induced Cardiomyopathy in Rats and Mice. A Modern View From the Perspective of the Pathophysiologist and the Clinician
Source: Front Pharmacol. 2021 Jun 3;12:670479. doi: 10.3389/fphar.2021.670479 (PMC8209419; doi:10.3389/fphar.2021.670479)
Supplement: Supplementary file 1 [file Image1.pdf]

Supplementary Material

1 Supplementary Figures

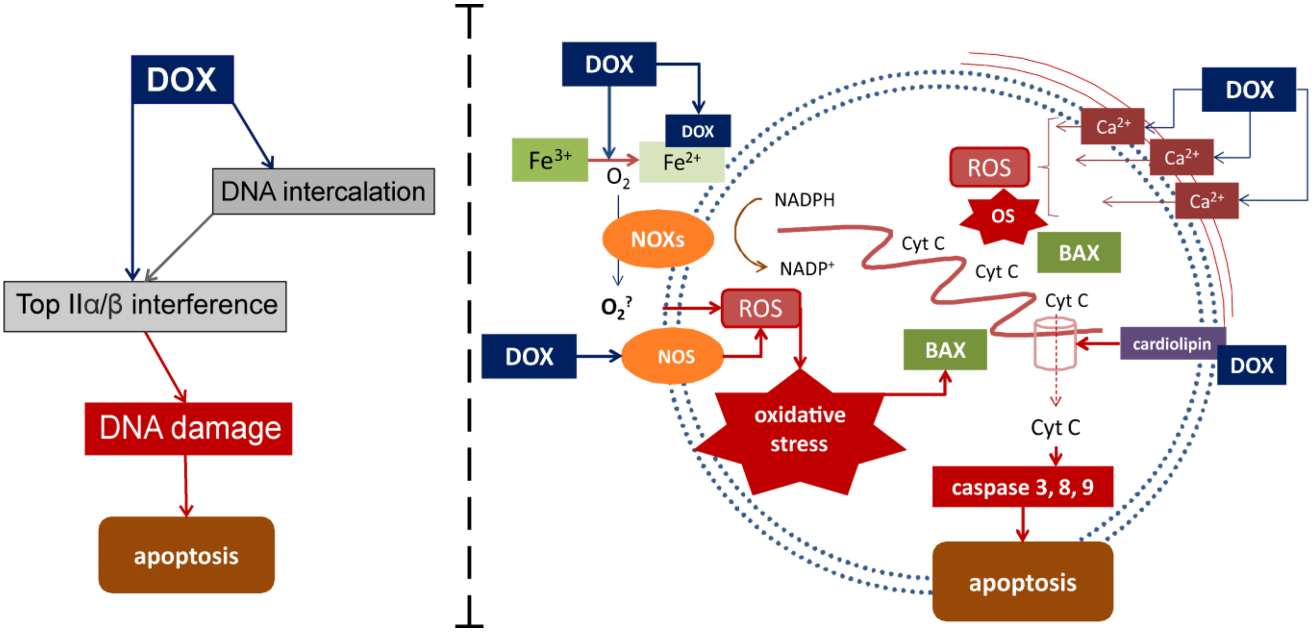

Figure 1. Mechanisms of Dox-induced cardiomyocyte damage. DOX targets Top2α/β. This leads to the suppression of the formation of the Top2-DNA cleavage complex, followed by the transcription arrest, which then results in the DNA damage and cell death. Dox also mediates apoptosis through interaction with Fe with subsequent active oxygen releasing and oxidative stress activation. Another way for oxidative stress activation is Dox-induced increasing of calcium ions releasing from the sarcoplasmic reticulum. Binding to cardiolipin Dox leads to mitochondrial dysfunction. Active cytochrome C releasing from mitochondrias to cytoplasm through the pores activates inner ways of apoptosis. BAX - Bcl-2-associated X protein, Cyt C - cytochrome C, DOX – doxorubicin, NOS - nitric oxide synthases, eNOS - endothelial NOS, NOXs - nicotinamide adenine dinucleotide phosphate oxidases, NADPH - nicotinamide adenine dinucleotide phosphate, ROS - reactive oxygen species.
